# Mild, moderate or severe lung injury after trauma: what are the early predictors?

**DOI:** 10.1186/1757-7241-23-S2-A8

**Published:** 2015-09-11

**Authors:** Ruslan Zinchenko, Elaine Cole, Simon Glasgow, Ross Davenport

**Affiliations:** 1 Centre for Trauma and Neurosciences, Queen Mary University of London, London, UK

## Background

Lung injury is a common complication following major trauma. Large volume transfusions, thoracic injury, pulmonary contusion, and hypo perfusion have previously been associated with its development [1]. However, early predictors of differing grades of lung injury following trauma are yet to be described. The objective of this study was to characterise the admission risk factors associated with differing severity of lung injury in trauma patients.

## Method

This study took place in an urban Major Trauma Centre. We performed a retrospective analysis of prospectively collected data from adult trauma patients (>15 years) enrolled to the ACIT2 study over a five-year period. ACIT2 is an on-going prospective study of 2,800 patients evaluating coagulation and inflammation in trauma [2]. Lung injury was defined using the Murray Lung Injury (MLI) criteria [3]. Mild-moderate lung injury (MMLI) was defined as a MLI score of 0.1-2.5, and severe lung injury (SLI) as a score of >2.5. Multivariate analysis was used to identify independent predictors of lung injury, using SPSS.

## Results

Of 812 patients enrolled into the ACIT2 study, 311 were admitted to critical care. Of these 145 (47%) developed MMLI, and 47 (15%) developed SLI. Time period to SLI was longer (Days 6 vs 4, p<0.01). Patients who developed SLI were more severely injured (ISS 33 vs 29, p<0.01), (AIS thorax 4 vs 3, p<0.01) and more shocked on admission (base deficit 4.9 vs 3, p=0.01). The transfusion requirements didn't influence the severity of lung injury. Independent risk factors associated with SLI were older age (OR 1.07, p<0.01) and injury severity (OR 1.14, p<0.01). The severity of lung injury was not associated with increased mortality rates.

## Conclusion

Older age and increased injury severity were independent predictors of severe lung injury in this cohort of patients. Severity of lung injury was not associated with mortality in this study.
